# Site‐Specific Load‐Induced Expansion of Sca‐1^+^Prrx1^+^ and Sca‐1^−^Prrx1^+^ Cells in Adult Mouse Long Bone Is Attenuated With Age

**DOI:** 10.1002/jbm4.10199

**Published:** 2019-07-30

**Authors:** Pamela Cabahug‐Zuckerman, Chao Liu, Cinyee Cai, Ian Mahaffey, Stephanie C Norman, Whitney Cole, Alesha B Castillo

**Affiliations:** ^1^ Department of Orthopaedic Surgery NYU Langone Health, New York University New York NY USA; ^2^ Department of Biomedical Engineering Tandon School of Engineering, New York University New York NY USA; ^3^ Rehabilitation Research and Development Veterans Affairs New York Harbor Healthcare System New York NY USA; ^4^ Rehabilitation Research and Development Veterans Affairs Palo Alto Healthcare System Palo Alto CA USA

**Keywords:** LOAD‐INDUCED BONE FORMATION, AGING, OSTEOGENESIS, SKELETAL STEM CELL, PRRX1

## Abstract

Aging is associated with significant bone loss and increased fracture risk, which has been attributed to a diminished response to anabolic mechanical loading. In adults, skeletal progenitors proliferate and differentiate into bone‐forming osteoblasts in response to increasing mechanical stimuli, though the effects of aging on this response are not well‐understood. Here we show that both adult and aged mice exhibit load‐induced periosteal bone formation, though the response is significantly attenuated with age. We also show that the acute response of adult bone to loading involves expansion of Sca‐1^+^Prrx1^+^ and Sca‐1^−^Prrx1^+^ cells in the periosteum. On the endosteal surface, loading enhances proliferation of both these cell populations, though the response is delayed by 2 days relative to the periosteal surface. In contrast to the periosteum and endosteum, the marrow does not exhibit increased proliferation of Sca‐1^+^Prrx1^+^ cells, but only of Sca‐1^−^Prrx1^+^ cells, underscoring fundamental differences in how the stem cell niche in distinct bone envelopes respond to mechanical stimuli. Notably, the proliferative response to loading is absent in aged bone even though there are similar baseline numbers of Prrx1 + cells in the periosteum and endosteum, suggesting that the proliferative capacity of progenitors is attenuated with age, and proliferation of the Sca‐1^+^Prrx1^+^ population is critical for load‐induced periosteal bone formation. These findings provide a basis for the development of novel therapeutics targeting these cell populations to enhance osteogenesis for overcoming age‐related bone loss. © 2019 The Authors. *JBMR Plus* published by Wiley Periodicals, Inc. on behalf of American Society for Bone and Mineral Research.

## Introduction

Bone adapts to its mechanical environment by optimizing its size and shape to meet mechanical demands.^1^, ^2^ The bone envelope (ie, periosteal, endosteal, trabecular) to which new bone is added in response to mechanical stimuli can have significant effects on overall bone strength and fracture risk.^3^ Loading in children and adults typically results in periosteal bone apposition, as well as maintenance of endosteal and trabecular bone.^4^, ^5^ These changes, particularly the addition of bone to the periosteal surface, contribute to greater bending strength.^6^, ^7^, ^8^, ^9^, ^10^ Thus, mechanical stimulation in the form of load‐bearing exercise (eg, walking, running, weightlifting) has long been a strategy to maintain bone mass and mechanical integrity of the skeleton throughout life.

Age‐related bone loss is caused by a negative bone balance characterized by reduced bone formation rates and increased endosteal and trabecular bone resorption.^11^, ^12^, ^13^ By mechanisms not yet fully understood, the ability of the aged skeleton to respond to increased mechanical stimulation diminishes,^4^ and this age‐related reduction in mechanoresponsiveness renders exercise less effective in building bone mass in the aging skeleton.^5^ Likewise in rodent models, in vivo exogenous mechanical loading leads to new bone formation and reduced bone resorption,^10^ and this response is attenuated with aging.^1^, ^11^, ^12^, ^13^, ^14^, ^15^ That is, though aged rodents are able to form new bone, the strain level required to activate new bone formation, also referred to as the “anabolic strain threshold,” is higher, and the amount of bone formed per unit strain, or “mechanoresponsiveness,” is diminished.^1^ The most recent data suggest that, in lieu of a strain threshold, both bone formation and resorption occur on a strain continuum with some specificity; that is, high strains lead to greater formation and reduced resorption and low strains lead to greater resorption and reduced formation.^15^ With aging, it appears that this strain specificity is disrupted so that both formation and resorption occur in regions of both high and low strain. Underlying mechanisms responsible for this age‐related change are unclear.

Load‐induced lamellar bone formation, as assessed using double fluorescent bone labeling, occurs on three distinct surfaces (periosteal, endosteal, and trabecular) and is likely driven by both the proliferation of resident osteoprogenitors and the recruitment of skeletal stem cells from the marrow.^16^ In fact, > 90% of osteoblasts at the periosteal surface of a loaded bone originate from proliferating cells, though their origin is unclear. Furthermore, the relative contribution of specific cell populations to load‐induced bone formation is virtually unknown.

Each bone surface is structurally different^17^ and consists of a cellular niche with its own inherent osteoprogenitor composition and function^18^, ^19^: the periosteum contains primarily bipotent osteochondral progenitors expressing paired related homeobox 1 (Prrx1),^20^, ^21^ Sca‐1, CD51, CD44, and the leptin receptor (LepR)^22^; the endosteum contains primarily osterix (Osx)^23^ and CD166^24^ positive cells; the trabecular compartment is encased in marrow, which contains osteoprogenitors that have been identified by various markers including LepR,^25^ CXCL12,^26^ Sca‐1,^24^, ^27^ Prrx1,^21^ CD166,^24^ PDGFRa,^27^ Nestin,^28^ NG2,^29^ and Gremlin 1.^30^

The comparative effects of loading on each of these envelopes, and by extension, on their resident osteoprogenitor populations, are not well‐understood. Furthermore, how aging affects each niche in the context of mechanical loading is virtually unknown. The age‐related response may involve reduced numbers of osteoprogenitor cells,^31^ their reduced proliferative and osteogenic capacity,^32^ and/or hindered stem cell recruitment from the marrow.^16^

The objectives of this study were to first demonstrate attenuated load‐induced bone formation in aged mice using our mechanical loading system, and then to evaluate age‐related changes in osteoprogenitor cell populations in tibial periosteum, endosteum, and marrow in response to exogenous mechanical loading. Skeletal multipotent mesenchymal stromal cells have been identified by various markers (reviewed in Kfoury and Scadden, 2015^33^); here we use stem cell antigen‐1 (Sca‐1) and paired related homeobox 1 (Prrx1) to identify subsets of skeletal progenitors^34^, ^35^ in adult and aged mouse bone. Sca‐1^+^Prrx1^+^ cells have been considered a more primitive osteogenic population because of their greater homogeneity and lower levels of Runx2 expression compared with Sca‐1^−^Prrx1^+^ cells; that is, Sca‐1 expression is reduced as Runx2 expression increases.^35^ However, Runx2 expression has not been assessed in this work; we therefore do not refer to either of these populations as being more primitive than the other. We hypothesized that aged mice would exhibit attenuated bone formation, as others have previously shown,^1^, ^2^, ^13^, ^15^, ^36^, ^37^ and that Sca‐1^+^Prrx1^+^ and Sca‐1^−^Prrx1^+^ progenitor cells would be fewer in number and exhibit a diminished proliferative response to mechanical loading.

## Materials and Methods

### Animals

Adult and aged WT female C57BL/6 mice were obtained from the Jackson Laboratory (Bar Harbor, ME, USA). All protocols were approved by the New York University Institutional Animal Care and Use Committee. Animals had access *ad libitum* to standard mouse chow and water. Adult and aged mice were weighed at the beginning and end of the study and were euthanized by asphyxiation and cervical dislocation prior to tissue harvest. The overall experimental plan, timeline, outcome measures, and volumes of interest examined are shown in Supplementary Fig. S1.

### Study 1: In vivo load‐induced bone formation and structural adaptation

#### Ex vivo load‐strain calibration procedure

Peak mechanical strains achieved on the periosteal surface of the tibia during in vivo axial compressive loading were estimated using a load‐strain calibration procedure as described previously.^38^ Sixteen‐week‐old (*n* = 5) and 52‐week‐old (*n* = 5) WT C57Bl/6 mice were euthanized by CO_2_ asphyxiation and cervical dislocation. Immediately after euthanasia, a longitudinal incision was made at the tibial midshaft, and the musculature was retracted exposing only the anteromedial diaphysis of the tibia. The periosteum was scraped off using a scalpel blade and cleaned with 70% ethanol. A 120 Ω single‐element strain gauge (EA‐06‐015DJ‐120; Vishay Measurements Group, Wendell, NC, USA) was glued to the surface with cyanoacrylate (M‐Bond 200; Vishay Measurements Group) centered approximately 3.75 mm proximal to the tibiofibular junction. Each gauge was conditioned with a 0.8 V bridge excitation voltage and amplified with a gain of 300 × using a signal conditioner (Model 2210; Vishay Measurements Group). The amplified analog gauge signals were digitized using an AD‐DA board (aISA‐A57; Adtek‐System Science, Kanagawa, Japan) and evaluated using an oscilloscope (Agilent Infiniium 54830B DSO; Agilent Technologies, Santa Clara, CA, USA). With the strain gauge voltage zeroed, each tibia was axially loaded using a mechanical loading system (Bose ElectroForce 3200; Bose Corporation, Minnetonka, MN, USA) at increasing load levels, beginning at 1 N and incrementally increasing by 1 N up to 5 N. The average peak‐to‐peak voltage was observed on the oscilloscope and recorded. Voltage data for each of the loading waveforms were converted to strain values using a conversion factor (1 V = 1000 με), which was confirmed by electronic shunt calibration of the measuring hardware and by calculated strains using an aluminum cantilever. Load‐strain calibration curves were not significantly different (Supplementary Fig. S2, see Results for details). Given that the cross‐sectional bone geometry can vary between the location at which strain gauges are placed in calibration animals and the location at which bone labels are analyzed in experimental animals, peak strains induced were estimated using the same sections for which bone formation rates were calculated. Applied load, flexural modulus, the distance from the periosteal surface to the neutral axis, and second moment of area were determined for each cross‐section, and periosteal strain was estimated using a predetermined constant, which was derived during the load‐strain calibration procedure, as described previously.^38^

#### In vivo axial compressive tibial loading

To assess effects of aging on load‐induced bone formation, 16‐week‐old (*n* = 8) and 52‐week‐old (*n* = 8) WT C57Bl/6 mice were subjected to axial compressive tibial loading for 2 weeks. All mice were euthanized 1 week after the last loading bout. Bone geometry and bone formation rates in loaded and control tibias were evaluated using µCT analyses and dynamic histomorphometry, respectively. Right tibias were subjected to cyclic axial compressive loading, while left tibias served as internal nonloaded controls. The loading regimen (5 N peak load; 120 cycles; 2 Hz; Monday, Wednesday, Friday for 2 weeks) was applied via load feedback using a 50‐lb load cell (Honeywell Sensotec, Columbus, OH, USA), while the animal was under general isoflurane anesthesia (Forane; Baxter International, Deerfield, IL, USA). These studies were load‐matched given that the load‐calibration curves were not significantly different between adult and aged mice (Supplementary Fig. S2). To assess new bone formation, all mice were injected with in vivo fluorochrome bone labels at 4 (calcein, 30 mg/kg, i.p.), 11 (alizarin, 50 mg/kg, i.p.), and 18 (calcein, 30 mg/kg, i.p.) days after the first day of loading. Mice were euthanized on day 20, and tibias were harvested for µCT scanning and dynamic histomorphometry.

#### Microcomputed tomography

Loaded and control tibias were scanned using a preclinical µCT scanner (VivaCT; Scanco Medical AG, Brüttisellen, Switzerland) at a 9‐μm isotropic voxel size with a tube voltage of 55 kVp, a 114 mA intensity, and a 300 ms integration time. Cortical geometry and trabecular microarchitecture were evaluated using the manufacturer's software and a constant global threshold CT value of 171, which represents a percentage of the CT value range based on the linear attenuation coefficient per the manufacturer's recommendations and preliminary studies comparing the original and segmented scans side‐by‐side to ensure segmentation accuracy. Cortical area (Ct.Ar, mm^2^), cortical thickness (Ct.Th, mm^2^), maximum moment of inertia (Imax, mm^4^), minimum moment of inertia (Imin, mm^4^), and polar moment of inertia (pMOI, mm^4^) were evaluated at cortical midshaft. A volume of interest starting at 0.25 mm below the growth plate and spanning 250 slices distally was identified using an automated selection routine supplied by the manufacturer. The VOI was evaluated for bone volume (BV/TV, %), trabecular number (Tb.N, 1/mm), trabecular thickness (Tb.Th, mm), trabecular spacing (Tb.Sp, mm), connectivity density (Conn.D, 1/mm^3^), and structure model index (SMI)^39^ in which a value of 0 represents plates, 3 represents rods, and 4 represents solid spheres.

#### Dynamic histomorphometry

After scanning, tibias were dehydrated in sequential ascending concentrations of ethanol (70%, 80%, 90%, and 100%) and embedded undecalcified in methylmethacrylate. Three 90‐μm‐thick sequential transverse sections were cut at the midshaft using an Isomet Precision Saw (Buehler Ltd., Lake Bluff, IL, USA). Two sections per tibia were analyzed at a magnification of 10 × using a Nikon TE‐2000/C1 confocal microscope (Nikon, Inc., Melville, NY, USA). Static histomorphometric variables on the periosteal surface were obtained using Image J software (NIH, Bethesda, MD, USA; https://imagej.nih.gov/ij/), and dynamic bone formation indices were calculated.^40^ Static variables were total bone perimeter (B.Pm, mm), single‐label perimeter (sL.Pm, mm), double‐label perimeter (dL.Pm, mm) and the interlabel width (Ir.L.Wi, μm). Dynamic variables calculated were mineralizing surface (MS/BS = 100 * [0.5 * sL. Pm + dL.Pm]/B.Pm, %), mineral apposition rate (MAR = dL.Ar/dL.Pm/days between labels, μm/day and bone formation rate (BFR/BS = MAR * [MS/BS] * 3.65, μm^3^/μm^2^/year).^40^ When only single labels were present, the mineral apposition rate was estimated as the minimum value observed in that specific experimental group.^40^ Based on a previously described protocol, load‐induced peak strains on the anteromedial periosteal surface were estimated using the same sections for which bone formation rates were calculated.^7^, ^38^, ^41^

### Study 2: Periosteal cell number and nuclear morphology

Load‐induced bone formation occurs preferentially on the periosteal surface in regions of high strain^10^ by activation of osteoprogenitors in the periosteum. We therefore evaluated the effects of aging on cells in the periosteum in terms of baseline periosteal cell number and nuclear morphology in tibias from 18‐week‐old (*n* = 3) and 53‐week‐old (*n* = 3) WT C57Bl/6 mice using confocal microscopy following whole‐mount 4,6‐diamidino‐2‐phenylindole (DAPI) staining.

#### Whole‐bone staining and imaging

Whole tibias were fixed with 4% paraformaldehyde (PFA) overnight at 4°C, then incubated in 0.15% Triton × 100 (Sigma‐Aldrich T8787; Sigma‐Aldrich, St. Louis, MO, USA) and 150 nM DAPI (Sigma‐Aldrich D9542) at 4°C for 72 hours. The anteromedial plane at midshaft was imaged using multiphoton imaging (Zeiss LSM 710 NLO; Zeiss, Inc., Thornwood, NY, USA), with a tunable Spectra‐Physics Mai Tai Ti‐Sapphire laser (Spectra‐Physics, Mountain View, CA, USA). A water immersion 20 × water‐immersion objective W Plan‐Apochromat (Zeiss, Inc.) 20 × /1.0 DIC M27 75 mm (numerical aperture [N.A.] 1.0) was used to acquire 100‐ to 130‐μm deep image stacks for both DAPI and second harmonic generation (SHG) signals. Volumes of interest (3 to 5 per animal) were selected using Imaris v7.4.2 (Bitplane USA, South Windsor, CT, USA) with dimenions of 420 width × 420 length × 10 depth μm. This VOI began at 5 μm superior to the edge of the SHG signal representing cortical bone collagen. From these planes, smaller (100 × 100) μm regions (*n* = 3 per plane) were analyzed for cell number based on DAPI nuclear staining. In addition, the area and aspect ratio of 60 nuclei per bone were quantified.

### Study 3: Acute cellular response to mechanical loading

To characterize the acute response of osteoprogenitors in the periosteal, endosteal, and marrow niches to mechanical stimulation, 16‐week‐old (*n* = 5 + 18) and 55‐ to 78‐week‐old (*n* = 3 + 11) WT C57Bl/6 mice were subjected to short‐term axial compressive tibial loading. Stem cell number and proliferative status were evaluated in loaded and control tibias using immunohistochemistry.

#### In vivo axial compressive tibial loading

##### Group 1

To study the acute effects of anabolic loading on the periosteal stem cell niche, 16‐week‐old (*n* = 5) and 55‐week‐old (*n* = 3) mice were subjected to 4 consecutive days of axial compressive loading (1200 με, 2 Hz, 120 cycles/day) of the right tibia while the left tibia served as an internal nonloaded control. Tibias were harvested at day 5 and analyzed for Prrx1 + and PCNA + cell number using thin‐section histology.

##### Group 2

To study the acute effects of loading on the periosteal, endosteal, and marrow stem cell niches, 16‐week‐old (*n* = 18) and 78‐week‐old (*n* = 11) mice were subjected to as many as 4 consecutive days of strain‐matched axial compression (1200 με, 2 Hz, 120 cycles/day). Mice were euthanized, and tibias harvested at day 2 (*n* = 6 adult, 4 aged), 4 (*n* = 6 adult, 4 aged) or 6 (*n* = 6 adult, 3 aged) after the first day of loading. Tibias were harvested and analyzed for Sca‐1 + , Prrx1 + , and Ki‐67 + cell numbers using deep‐tissue immunohistochemistry.

#### Thin‐section immunostaining, imaging, and quantification

Tibias were fixed in 4% PFA overnight at 4°C, decalcified with 19% EDTA pH = 7.4 for 21 days at 4°C, and paraffin embedded. Transverse 5‐μm sections from the tibial midshaft were processed by deparaffinization with Citrisolve (Fisher Scientific 04‐355‐121; Fisher Scientific, Pittsburgh, PA, USA) and rehydration in decreasing gradients of ethanol. Sections underwent heat‐activated antigen retrieval (IHC‐Tek Antigen Retrieval Solution #IW‐1100; IHC World, Woodstock, MD, USA) prior to incubation in 3% H_2_O_2_ to quench endogenous peroxidases. Sections were then incubated in a second antigen retrieval solution (Ficin, Thermo Scientific #003007; Thermo Fisher Scientific, Waltham, MA, USA) at room temperature before being blocked with 1% IgG/albumin and incubation in primary rabbit anti‐PCNA IgG (1:200 dilution, Cell Signaling Technologies #13110; Cell Signaling Technology, Danvers, MA, USA) overnight at 4°C. Anti‐PCNA was detected with a secondary goat anti‐rabbit IgG linked with biotin (Abcam ab6720; Abcam, Cambridge, MA, USA) followed by horseradish peroxidase‐ (HRP‐) streptavidin conjugate (Jackson ImmunoResearch 016‐030‐084; Jackson ImmunoResearch, West Grove, PA, USA), which was developed with 3,3’‐diaminobenzidine (DAB; Thermo Scientific 34002) detection system. Sections were counterstained with hematoxylin (Sigma‐Aldrich HT1079) to detect nuclei. Prrx1 was detected (Anti‐Prrx1, Abcam ab211292) using the same protocol described for HRP chromogen detection. Three sections per bone per animal were imaged at 40 × (Leica DM5500; Leica, Wetzlar, Germany) and used to calculate Prrx1 + cell number per periosteal perimeter (Pm; Prrx1 + cells/Pm) and the percentage of total cells that were PCNA + per periosteal perimeter (% PCNA + cells/Pm). The total number of PCNA + and Prrx1 + cells at the periosteal region were quantified and normalized per total number of cells per perimeter of cortical bone underneath the periosteum using ImageJ.

#### Thick‐section preparation and immunostaining

Tibias were fixed in 4% PFA at 4°C for 4 hours, decalcified in 0.5 M EDTA pH = 7.4 for 48 hours at 4°C, and cryoprotected in 20% sucrose prior to embedding in 22‐oxacalcitrol compound. Serially transverse 150‐µm‐thick sections were prepared at a region measuring 50% of the total tibia length. Sections were rinsed in PBS 3 times for 10 min, permeabilized with 0.3% Triton X, blocked with 1% BSA for 30 min at room temperature. Sections were incubated in a cocktail of blocking solution and primary antibodies against Sca‐1 (rat, eBioscience Cat#14‐5981‐82; eBioscience, Santa Clara, CA, USA), Prrx1 (rabbit, Abcam ab211292), Ki‐67 (sheep, R&D AD7649‐SP; R&D Systems, Minneapolis, MN, USA, and CD31 (goat, Santa Cruz sc1506; Santa Cruz Biotechnology, Santa Cruz, CA, USA) in 1:100 dilutions at 4°C for 18 hours. Nonimmune species‐specific IgG or serum was used for negative controls. Sections were then incubated in a cocktail of secondary antibodies (1:400) consisting of anti‐rat Alexa Fluor 594 (Fisher Scientific A‐21209), anti‐rabbit Alexa Fluor 488 (Fisher Scientific A‐21206), anti‐sheep Alexa Fluor 405 (Abcam ab75676), and anti‐goat Alexa Fluor 647 (Jackson ImmunoResearch 712‐606‐150) in PBS for 2 hours at room temperature (Supplementary Tables S1 to S3). Sections were mounted onto glass slides using an aqueous mounting medium (Sigma‐Aldrich F4680) for imaging using confocal microscopy.

#### Confocal microscopy

Sections were imaged using a Zeiss LSM710 confocal microscope with a 20× water immersion objective W Plan‐Apochromat 20 × /1.0 DIC M27 75 mm (N.A. 1.0), with a pinhole size of 32.6 µm, which range 1 to 1.2 Airy unit for all channels. To avoid crosstalk, all four channels were split into two tracks: A405 and A594, A488 and A633. Acquisition parameters for each antibody are provided in Supplementary Table S4. Monochromatic lasers (405 nm, 488 nm, 543 nm, 633 nm) were used for confocal microscopy with detection filters: 420 to 460 nm, 510 to 540 nm, 610 to 650 nm, and 660 to 700 nm, respectively. Gain for all lasers was 650; laser intensity: 5%, 7%, 30%, and 15%, respectively. Combinations of up to four fluorescent dyes (Alexa Fluor 405, Alexa Fluor 488, Alexa Fluor 594, Alexa Fluor 633) were acquired using separate channels. Detection filters matched the spectral properties of fluorochromes. Image stacks were stitched using an automated tiling routine (Rectangular Grid Stitched Image) provided by the manufacturer (Zen 2012 SP1 v8.1.9.484; Zeiss, Inc.) The volumes of interest imaged were: (1) the periosteum, which included the entire extent at the anteromedial aspect of the tibia as noted by cellular distribution of embedded osteocytes and extended 40 μm above cortical bone; (2) the endosteum, which was located at the opposing side of the periosteal volume of interest and encompassed the volume between the edge of cortical bone and 20 µm distally; and (3) the marrow, which was the entire volume of the marrow minus the endosteal region.

#### Image processing, cell segmentation, and cell quantification

Image stacks were rendered into 3D volumes and analyzed using Imaris v7.4.2 (Bitplane USA). Large regions were imaged in tiles, which were then stitched together using an automated tiling routine (Rectangular Grid Stitched Image) provided by the manufacturer (Zen 2012 SP1 v8.1.9.484; Zeiss, Inc.) Image intensities were used to categorize individual voxels and determine “their correspondence to segmented” structures within bone and bone marrow. These results were then used to digitally reconstruct cells and structures as objects in 3D space. Objects positively stained for Sca‐1, Prrx1, CD31, and Ki‐67 were detected automatically using intensity values based on predefined dimensions for expected whole‐cell and nuclear size (Supplementary Table S5). The automated routine for identifying and counting cells was compared with a manual count of the same image datasets for validation (Supplementary Tables S6 and S7, Supplementary Fig. S3). As a control for nonspecific binding of antibodies in our thick‐section immunostaining, we analyzed control and loaded tibias from Prrx1‐CreER‐eGFP (Jackson Labs #029211) transgenic mice (Supplementary Fig. S4). For the membrane‐bound proteins Sca‐1 and CD31 (labeled with Alexa Fluor 546 and Alexa Fluor 633, respectively), the allowed range of values was 200 to 1200 µm^3^ for volume (V) and 6 to 10 µm for diameter (D). For the nuclear proteins Prrx1 and Ki‐67 (labeled with Alexa Fluor 488 and Alexa Fluor 405, respectively), the allowed range of values was 125 to 1000 µm^3^ for V and 5 to 6 µm for D. Coexpression of individual markers was designated when the centroid of individual rendered cells was within 2 µm. Numbers of specific cell populations (Sca‐1^+^Prrx1^+^ Ki‐67 + /‒; Sca‐1^−^Prrx1^+^ Ki‐67 + /‒) were then calculated using blinded manual counts with one observer. For all channels, the allowed range of sphericity, or roundness, was 0.3 to 0.95, where sphericity = 1 for a sphere, and sphericity <1 for a shape departing a sphere. The volumes of interest (periosteum, endosteum, and marrow) and identification of individual cells are shown in Supplementary Fig. S5.

#### Data analysis

Data are reported as mean ± SD. Prism 6 Statistical Software (GraphPad Software, Inc., La Jolla, CA, USA) was used for all analyses. Differences in the slopes and intercepts of the load‐strain calibration curves were tested using linear regression analysis. Differences in cortical bone geometric properties, bone‐formation parameters, and trabecular bone microarchitecture parameters were tested for significance using a repeated measures two‐way ANOVA with a Tukey's correction for multiple comparisons and with age and loading as the main factors. Differences in periosteal‐resident cell number, nuclear area, and nuclear aspect ratio acquired from whole‐mount DAPI‐stained bones were tested for significance using a parametric, two‐tailed unpaired Student's *t* test with 95% confidence level between adult and aged values. Differences in periosteal‐resident Prrx1 + and PCNA + cell number acquired from thin‐section immunohistochemistry, and differences in cell number acquired from deep‐tissue imaging were tested for significance using a two‐way ANOVA with a Tukey's correction for multiple comparisons with age and loading as the main factors. Difference in cell number for individual experimental groups (ie, adult nonloaded, adult loaded, aged nonloaded, aged loaded) at timepoints 2, 4, and 6 were tested for significance using a one‐way ANOVA with time as the main factor. Differences in cell number between the periosteum and endosteum for individual experimental groups were tested for significance using a parametric, two‐tailed unpaired Student's *t* test with 95% confidence level. Significance for all tests was set at *p* < 0.05.

## Results

### Attenuated load‐induced periosteal bone formation in aged tibias

**Figure 1:**
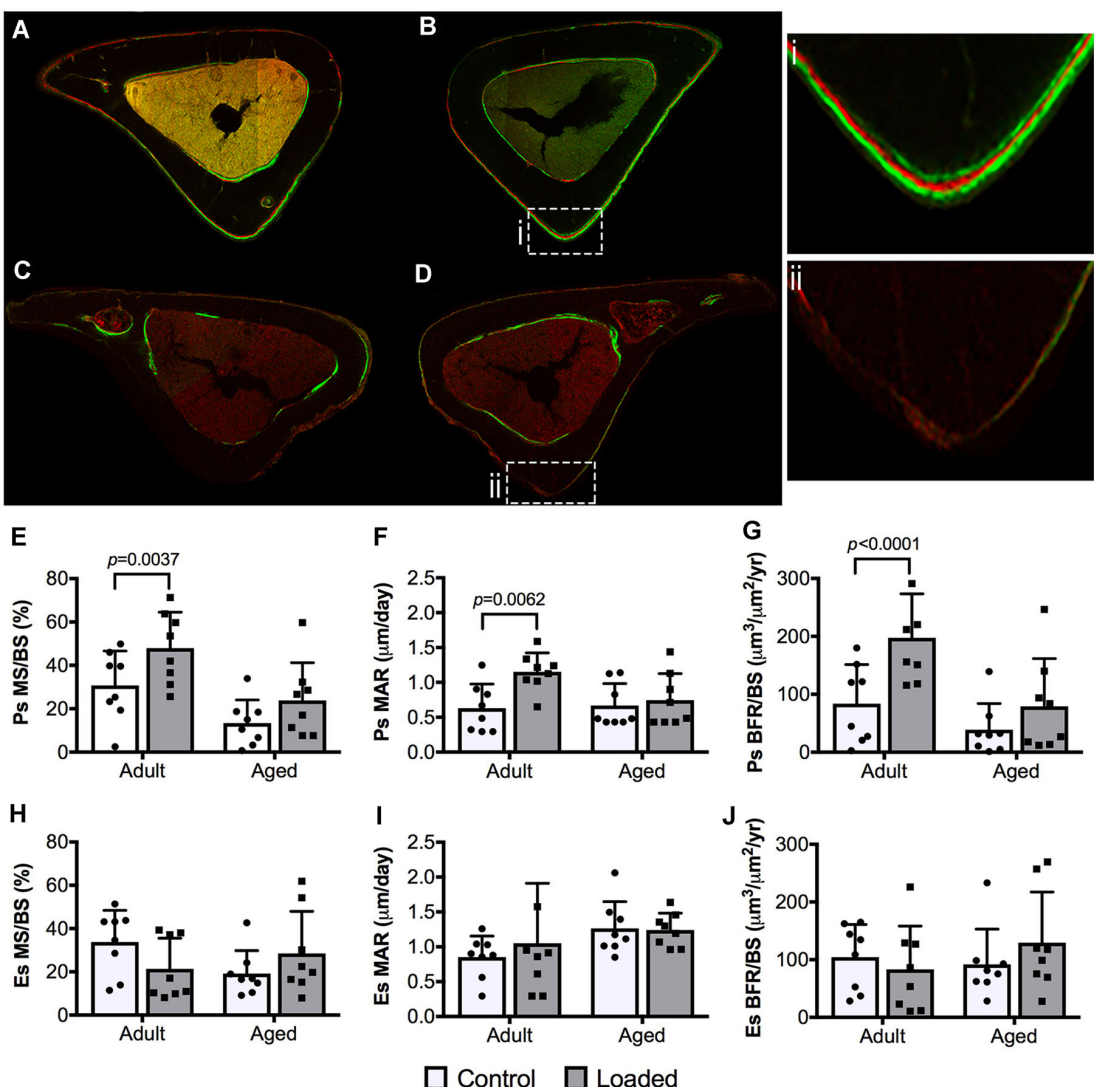
Representative images of transverse sections at tibial midshaft showing fluorochrome bone labels (calcein green and Alizarin Red) on bone surfaces in adult and aged control and loaded mouse tibias: (*A*) 16‐week‐old control tibia; (*B*) 16‐week‐old loaded tibia; (*B*.i) fluorochrome triple labels at the periosteal (Ps) surface; (*C*) 52‐week‐old control tibia; (*D*) 52‐week‐old loaded tibia; (*D*.ii) Ps surface void of fluorochrome labels. (*E,H*) Mineralizing surface (MS/BS, %), (*F,I*) mineral apposition rate (MAR, μm/day), and (*G,J*) bone formation rates (BFR/BS, μm^3^/μm^2^/year) at the Ps and endosteal (Es) surfaces. Data were analyzed using a two‐way repeated measures ANOVA with a Šídák post hoc analysis for multiple comparisons at α = 0.05.

The slopes of the load‐calibration curves were not significantly different between adult and aged mice (*p* = 0.450; Supplementary Fig. S2). The measured mean peak periosteal strains in the mice used for calibration were 1145 ± 166 μm and 925 ± 205 μm on the anteromedial aspect of the tibia in adult and aged mice, respectively. To demonstrate a reduction in load‐induced bone formation using our loading system, adult and aged mice were subjected to in vivo tibial axial compressive loading.^42^ Following a 2‐week load‐matched loading protocol, adult mice exhibited significantly greater periosteal bone formation rates (*p* < 0.0001) in loaded relative to control tibias (Fig. 1). This difference was caused by the combined effect of increases in both mineralizing surface (*p* = 0.0037; Fig. 1 *E*) and mineral apposition rate (*p* = 0.0062; Fig. 1 *F*). In contrast, aged mice did not exhibit a load‐induced increase in periosteal bone‐formation rates. Loading did not affect endosteal bone formation in either age group (Fig. 1 *H–J*).

### Loading alters cortical bone geometry, but not trabecular microarchitecture in adult and aged mice

Given that mechanical loading regulates both bone mass and geometry in healthy young bone, we investigated the extent to which aged mice responded similarly. Baseline cortical thickness in nonloaded control tibias was significantly lower in aged versus adult mice (*p* < 0.0001; Table 1. Following a 2‐week in vivo loading protocol, loaded tibias in adult mice exhibited significantly greater cortical area (*p* = 0.0005), Imax (*p* = 0.0225), Imin (*p* = 0.0163), and pMOI (*p* = 0.0044) relative to nonloaded controls. In aged mice, Imax (*p* = 0.004) and pMOI (*p* = 0.0045) were significantly greater in loaded versus nonloaded controls. Baseline trabecular bone volume (*p* = 0.002) and number (*p* = 0.0007) in nonloaded tibias were significantly lower in aged versus adult mice. Loading did not affect trabecular microarchitecture in either age group.

**Table 1:** Cortical Geometry and Trabecular Microarchitecture in Adult and Aged Control and Loaded Tibias by µCT

	*Adult control (n = 5)*	*Adult loaded (n = 5)*	*Aged control (n = 8)*	*Aged loaded (n = 8)*
Cortical geometry				
Cortical area (mm^2^)	0.741 ± 0.028	0.775 ± 0.019^a^	0.698 ± 0.037	0.698 ± 0.035
Total area (mm^2^)	1.144 ± 0.059	1.202 ± 0.019	1.254 ± 0.053	1.307 ± 0.092
Marrow area (mm^2^)	0.0045 ± 0.0011	0.0042 ± 0.0021	0.0047 ± 0.0017	0.0048 ± 0.0008
Cortical thickness (mm)	0.2264 ± 0.0040	0.2306 ± 0.0051	0.1840 ± 0.0145^b^	0.1919 ± 0.0089
Imax (mm^4^)	0.1290 ± 0.0069	0.1411 ± 0.0075^c^	0.1432 ± 0.0186	0.1559 ± 0.0206^d^
Imin (mm^4^)	0.0713 ± 0.0105	0.0799 ± 0.0053^e^	0.0794 ± 0.0066	0.0838 ± 0.0088
pMOI (mm^4^)	0.2011 ± 0.0128	0.2201 ± 0.0075^f^	0.2236 ± 0.0232	0.2386 ± 0.0272^g^
Trabecular microarchitecture				
BV/TV (%)	2.992 ± 1.002	2.866 ± 0.818	0.894 ± 0.683^h^	0.826 ± 0.642
Trabecular number (1/mm)	2.407 ± 0.187	2.295 ± 0.206	1.285 ± 0.337^b^	1.298 ± 0.295
Trabecular thickness (mm)	0.0461 ± 0.0029	0.0448 ± 0.0033	0.0550 ± 0.0094	0.0532 ± 0.0097
Trabecular spacing (mm)	0.4180 ± 0.0364	0.4444 ± 0.0395	0.8453 ± 0.2469^i^	0.8190 ± 0.1907
Connectivity density (1/mm^3^)	7.145 ± 5.043	6.963 ± 5.449	2.298 ± 5.294	1.768 ± 3.773
SMI^c^	3.479 ± 0.335	3.356 ± 0.223	3.119 ± 0.305	2.980 ± 0.521

Imax = maximum area moment of inertia; Imin = minimum area moment of inertia; pMOI = polar moment of inertia; BV/TV = bone volume/total volume; SMI = structure model index.

Loaded versus age‐matched controls: ^a^ *p* = 0.0005; ^c^ *p* = 0.0225; ^d^ *p* = 0.0040; ^e^ *p =* 0.0163; ^f^ *p* = 0.0044; ^g^ *p* = 0.0045.

Aged versus adult controls: ^b^ *p* < 0.0001; ^h^ *p* = 0.002; ^i^ *p* = 0.0007.

### Reduced cell number and altered nuclear morphology in aged periosteum

**Figure 2:**
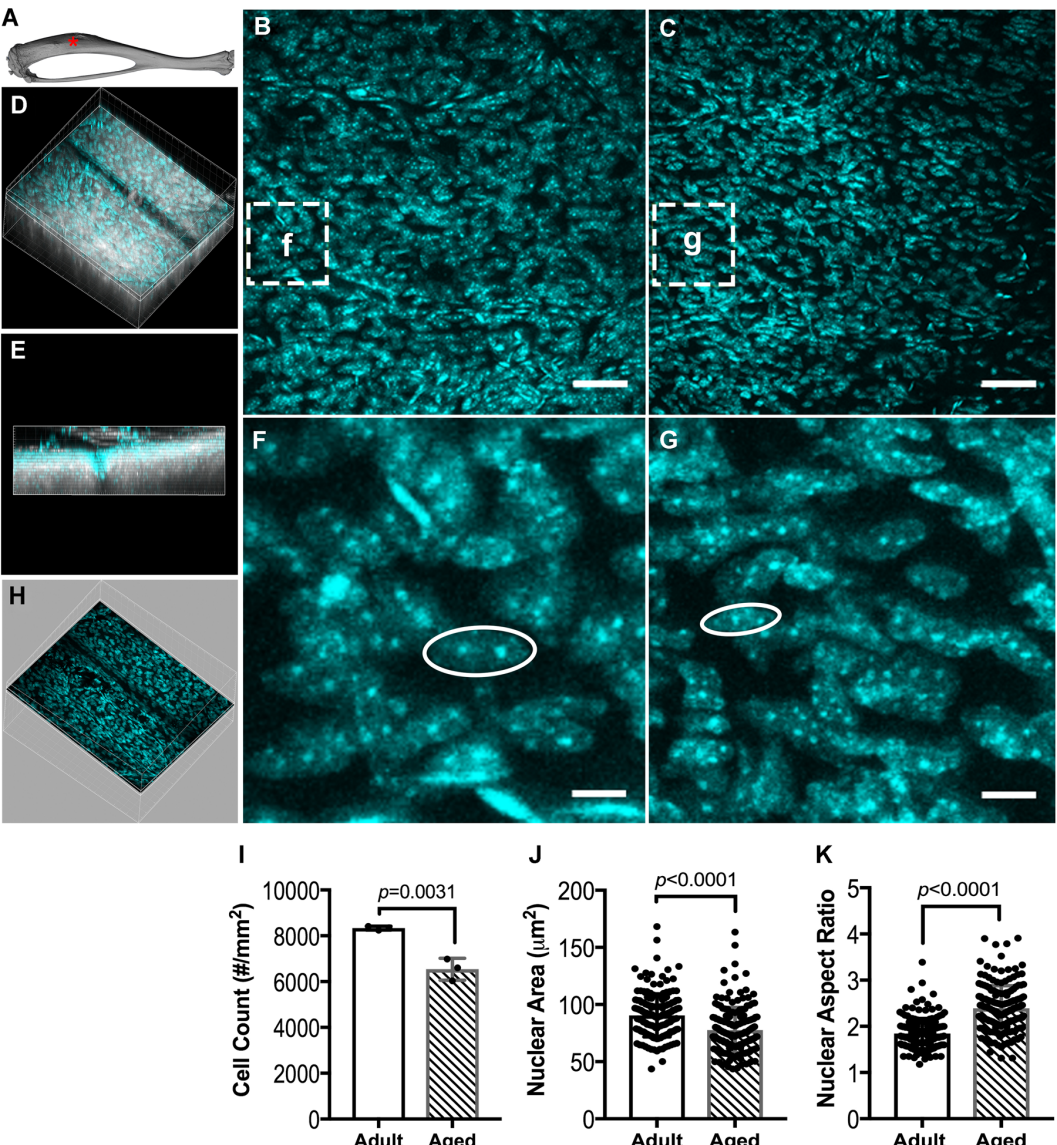
Periosteal DAPI staining of the (*A*) tibial anteromedial region (red asterisk). Representative z‐projection images of (*B*) adult and (*C*) aged periosteal cells, derived from periosteal volumes of interest. (*D*) Example of an image stack volume showing second harmonic generation (SHG, white) of cortical bone and DAPI stained cells (cyan), with (*E*) the corresponding side view. Magnified images of DAPI stained periosteal cells of (*F*) adult and (*G*) aged mice. Periosteal volume of interest (*H*) was separated from the raw image volume using SHG to distinguish cortical bone cells from periosteal cells. (*I*) Cell number, (*J*) nuclear area, and (*K*) nuclear aspect ratio of DAPI stained cells in adult and aged periosteum. Data are presented as mean ± SD. Scale bars in *B*, *C* = 50 µm. Scale bars in *F*, *G* = 5 µm.

We next investigated a potential basis for diminished osteogenic capacity in aged bone by assessing total periosteal cell number and nuclear morphology in the anteromedial aspect of tibia (Fig. 2 *A–G*), an indication of cell function.^43^, ^44^ Periosteal cell number (*p* = 0.0031) and nuclear area (*p* < 0.0001) were 27% and 17% lower, respectively, in aged versus adult mice (Fig. 2 *I,J*). The nuclear aspect ratio, which is the ratio of the major to minor axes, was 23% greater (*p* < 0.0001) in aged versus adult mice (Fig. 2 *K*).

### Mechanical loading activates proliferation of periosteal‐resident Sca‐1^+^Prrx1^+^ and Sca‐1^−^Prrx1^+^ cells in adult, but not aged bone

**Figure 3:**
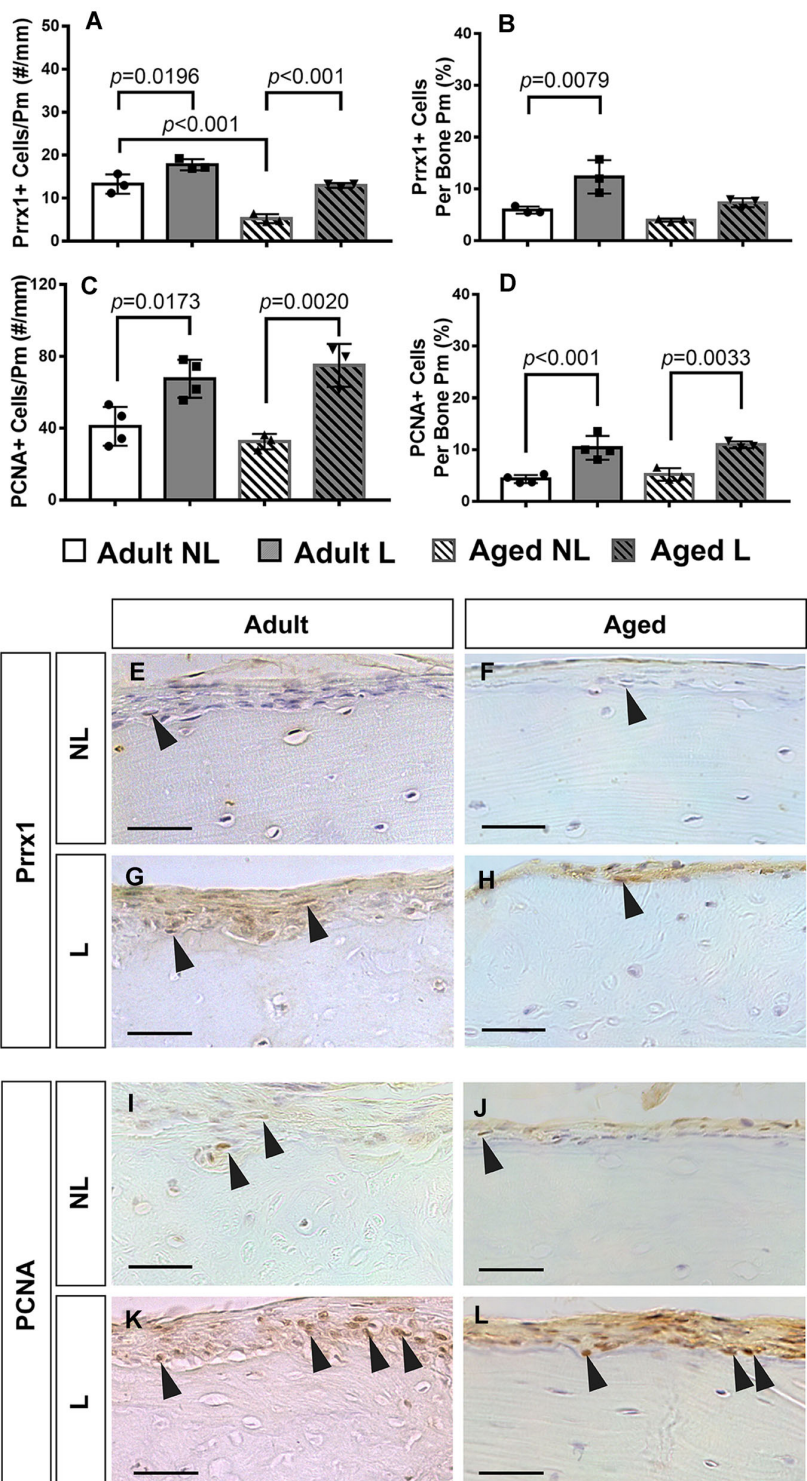
Quantification of Prrx1 + and PCNA + periosteal cells from immunohistochemical staining of thin paraffin‐embedded tissue sections from adult and aged nonloaded (NL) and loaded (L) tibias. (*A*) Prrx1 + cell number normalized to periosteal perimeter and (*B*) as the percentage of total cells. (*C*) PCNA + proliferating cell number normalized to periosteal perimeter and (*D*) the percentage of total cells. Representative images of the periosteum stained for Prrx1 (*E–H*) and PCNA (*I–L*) for all experimental groups. Black arrows denote positively stained cells. Scale bars = 50 µm.

To gain insight into the effects of aging on the response to mechanical loading of periosteal‐resident cells, Prrx1 + and PCNA + cells were quantified in thin transverse sections from strain‐matched loaded tibias in adult and aged mice. In both adult and aged mice, loading resulted in an increase in the total number of Prrx1 + cells, relative to their respective nonloaded controls (Fig. 3 *A*). Prrx1 + cell number in nonloaded control tibias was significantly lower in aged versus adult mice (*p* < 0.001). When Prrx1 + cell number was normalized by total cells counted rather than bone perimeter, the load‐induced increase in adult, but not aged mice was significant (*p* = 0.0079; Fig. 3 *B*). Loading resulted in significant increases in the number of proliferating PCNA + cells in both adult (*p* = 0.0173) and aged (*p* = 0.0020) mice when compared with their nonloaded controls (Fig. 3 *C*). These load‐induced differences persisted when PCNA + cell number was normalized by the total number of cells counted (Fig. 3 *D*). No differences in proliferation were observed in nonloaded control tibias between the two age groups (representative images are shown in Fig. 3 *E‐L*).

**Figure 4:**
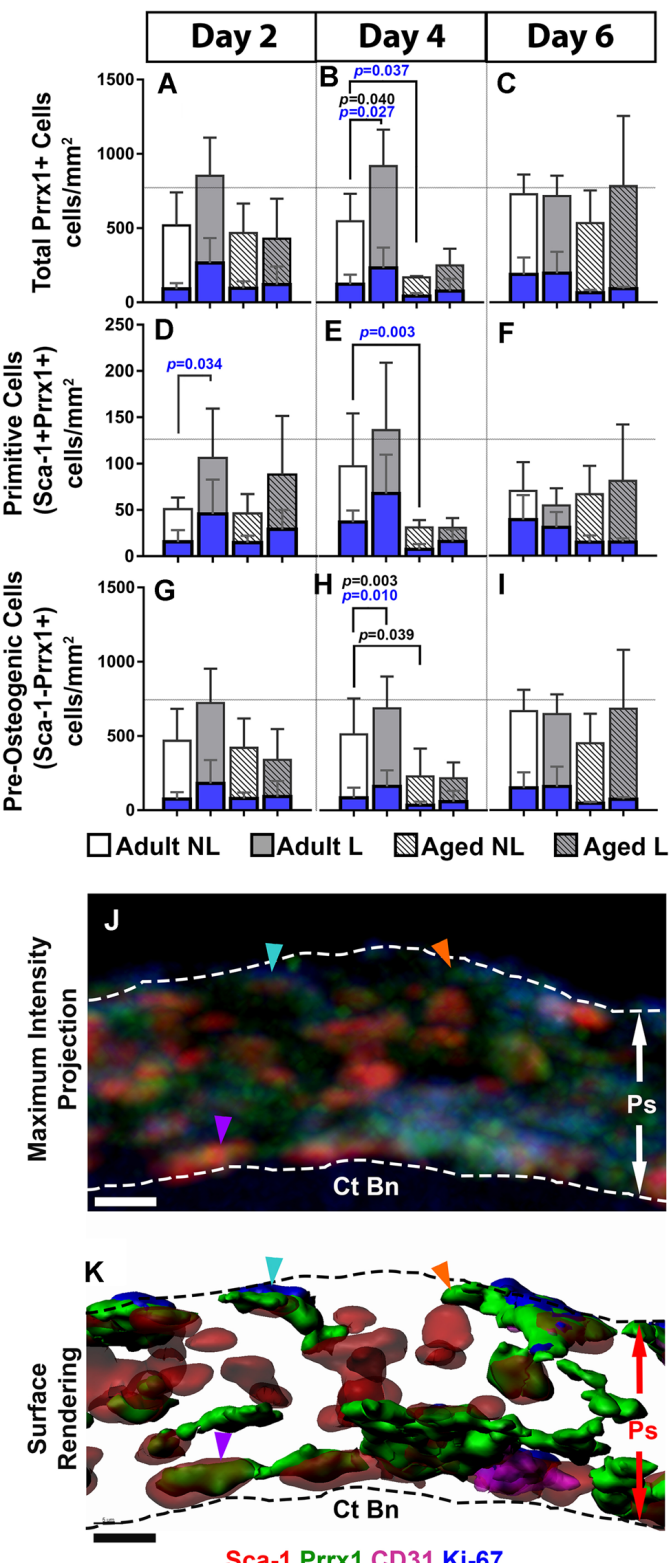
Quantification of periosteal‐resident stem cell populations in adult and aged nonloaded (NL) and loaded (L) tibias using deep‐tissue immunohistochemistry. Total cell numbers are represented by white and gray bars, and proliferating cell number within each of these populations are represented by blue bars. (*A–C*) Total Prrx1 + cells are divided into (*D–F*) Sca‐1^+^Prrx1^+^ and (*G–I*) Sca‐1^−^Prrx1^+^ subgroups at days 2, 4, and 6. Representative image of the periosteum (Ps) from adult loaded bone displayed as a (*J*) maximum intensity projection and the corresponding (*K*) surface rendering showing Sca‐1 + (red), Prrx1 + (green), CD31 + (magenta), and Ki‐67 + (blue) cells atop cortical bone (Ct Bn) and demarcated by dotted lines. The periosteum contains resting Sca‐1^−^Prrx1^+^ Ki‐67– (orange arrow) and dividing Sca‐1^−^Prrx1^+^ Ki‐67 + (aqua arrow) cells, and resting Sca‐1^+^Prrx1^+^ cells (purple arrow). Note the presence of CD31 + cells in magenta are proximal to Prrx1 + cells. Significant differences in total cell number are indicated by *p* values shown in black. Significant differences in proliferating cell number are indicated by *p* values shown in blue. Scale bars = 5 µm.

In a second experiment, 3D deep‐tissue imaging was used to quantify total Prrx1 + cells (Fig. 4 *A‐C*) and to distinguish between Sca‐1^+^Prrx1^+^ (Fig. 4 *D‐F*) and Sca‐1^−^Prrx1^+^ (Fig. *4G‐I*) cells in this population at 2, 4, and 6 days following the first day of a 4‐day loading protocol. Their proliferative status was assessed by Ki‐67 + staining. First, the periosteum contains greater numbers of Sca‐1^−^Prrx1^+^ versus Sca‐1^+^Prrx1^+^ cells. The total and proliferating numbers of Prrx1 + cells were significantly greater in adult, but not aged bone at day 4 (Fig. 4 *B*). In addition, the total and proliferating numbers of Prrx1 + cells were significantly lower in aged versus adult bone. When divided into subpopulations, proliferating Sca‐1^+^Prrx1^+^ cells were significantly greater in loaded versus control bones in adult, but not aged mice at day 2 (Fig. 4 *D*). A similar increase was observed in Sca‐1^−^Prrx1^+^ cells in adult, but not aged bone at day 4 (Fig. 4 *H*; representative images are shown in Fig. 4 *J‐K*). Together these data suggest that loading preferentially targets the periosteal‐resident Sca‐1^+^Prrx1^+^ cell population during the acute response phase (by day 2), and that this effect diminishes with age.

### Endocortical‐resident total and proliferating Prrx1 + cells increase in adult loaded tibias over time

**Figure 5:**
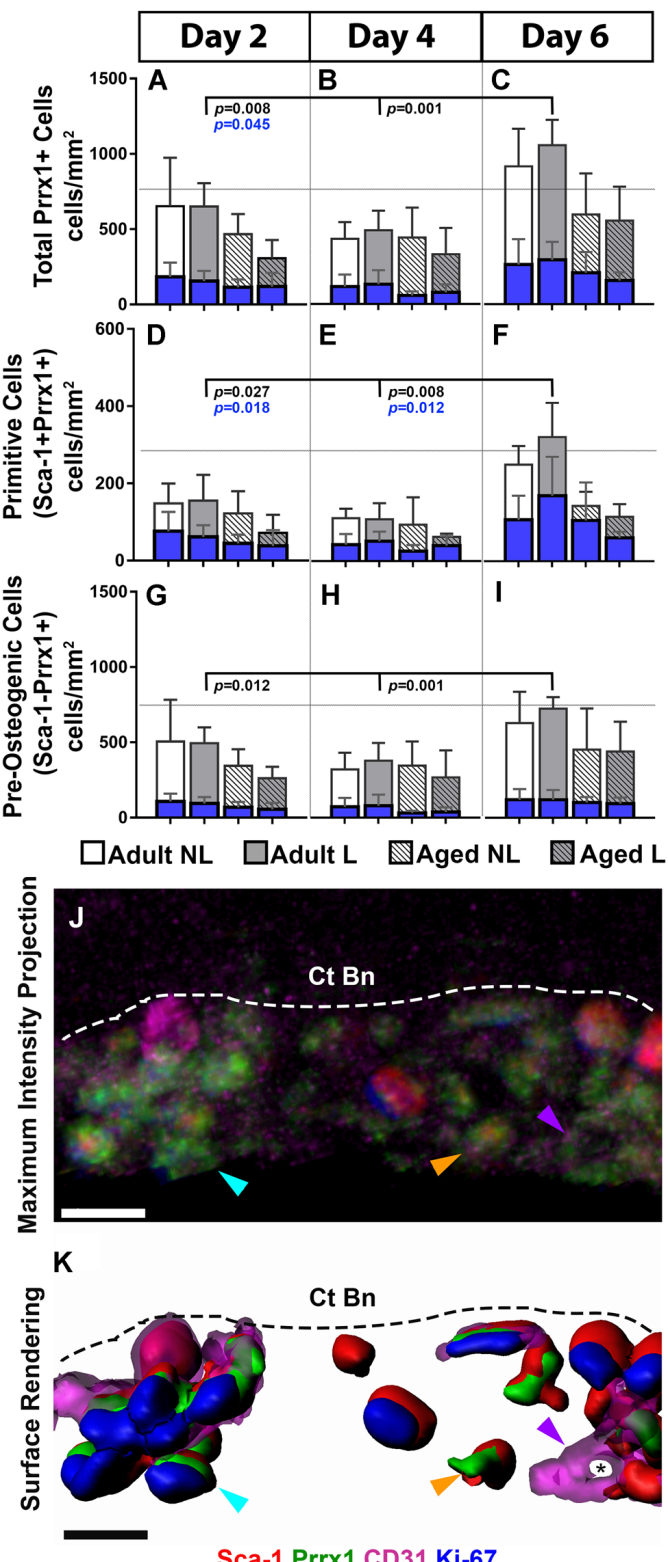
Quantification of endosteal‐resident cell populations in adult and aged nonloaded (NL) and loaded (L) tibias using deep‐tissue immunohistochemistry. Total cell numbers are represented by white and gray bars, and proliferating cell number within each of these populations are represented by blue bars. (*A–C*) Total Prrx1 + cells are divided into (*D–F*) Sca‐1^+^Prrx1^+^ and (*G–I*) Sca‐1^−^Prrx1^+^ groups at days 2, 4, and 6. Representative image of the endosteum from adult loaded bone displayed as a (*J*) maximum intensity projection and the corresponding (*K*) surface rendering showing Sca‐1 + (red), Prrx1 + (green), Ki‐67 + (blue), and CD31 + (magenta) cells adjacent to cortical bone (Ct Bn) as demarcated by a dotted line. The endosteum contains resting Sca‐1^+^Prrx1^+^ Ki‐67– cells (orange arrow), proliferating Sca‐1^+^Prrx1^+^ Ki‐67 + cells (aqua arrow) and CD31 + endothelial cells of an existing lumen (black asterisk). Significant differences in total cell number are indicated by *p* values shown in black. Significant differences in proliferating cell number are indicated by *p* values shown in blue. Ct Bn = cortical bone. Scale bars = 5 µm.

The endosteum, like the periosteum, contains greater numbers of Sca‐1^−^Prrx1^+^ versus Sca‐1^+^Prrx1^+^ cells. At the endocortical surface, no significant differences in the number of total or proliferating Prrx1 + cells between loaded and nonloaded bones were detected at any one time point examined (Fig. 5). However, at day 6 there were significant increases in total Prrx1 + cell number in loaded adult tibias (Fig. 5 *C*) relative to days 2 (*p* = 0.008) and 4 (*p* = 0.001; Fig. 5 *A–C*). In addition, there was a significant increase in proliferating Prrx1 + cells in loaded adult tibias compared with day 2 (*p* = 0.045; Fig. 5 *A,C*). When Prrx1 + cells were split into subcategories, an increase in the number of total Sca‐1^+^Prrx1^+^ and Sca‐1^−^Prrx1^+^ cells on day 6 relative to days 2 and 4 was observed (Fig. 5 *D–I*); however, only Sca‐1^+^Prrx1^+^ cells showed a significant increase in proliferation on day 6 relative to days 2 and 4 (Fig. 5 *D–F*). At day 6, after 2 days without loading, the cell population observed to increase was the Sca‐1^−^Prrx1^+^ cells. A comparison of the periosteal and endosteal responses to loading showed that in control bone at day 2, the total (*p* = 0.0412) and proliferating (*p* = 0.0164) Sca‐1^+^Prrx1^+^ cell numbers were significantly greater at the endocortical surface in adult bone, with similar trends observed in aged bone (Supplementary Table S7). These data suggest that loading preferentially targets the endosteal‐resident Sca‐1^+^Prrx1^+^ cell population, with a slight delay in response relative to the periosteum, and that this effect diminishes with age.

### Mechanical loading preferentially activates the Sca‐1^−^Prrx1^+^ cell population in the marrow

**Figure 6:**
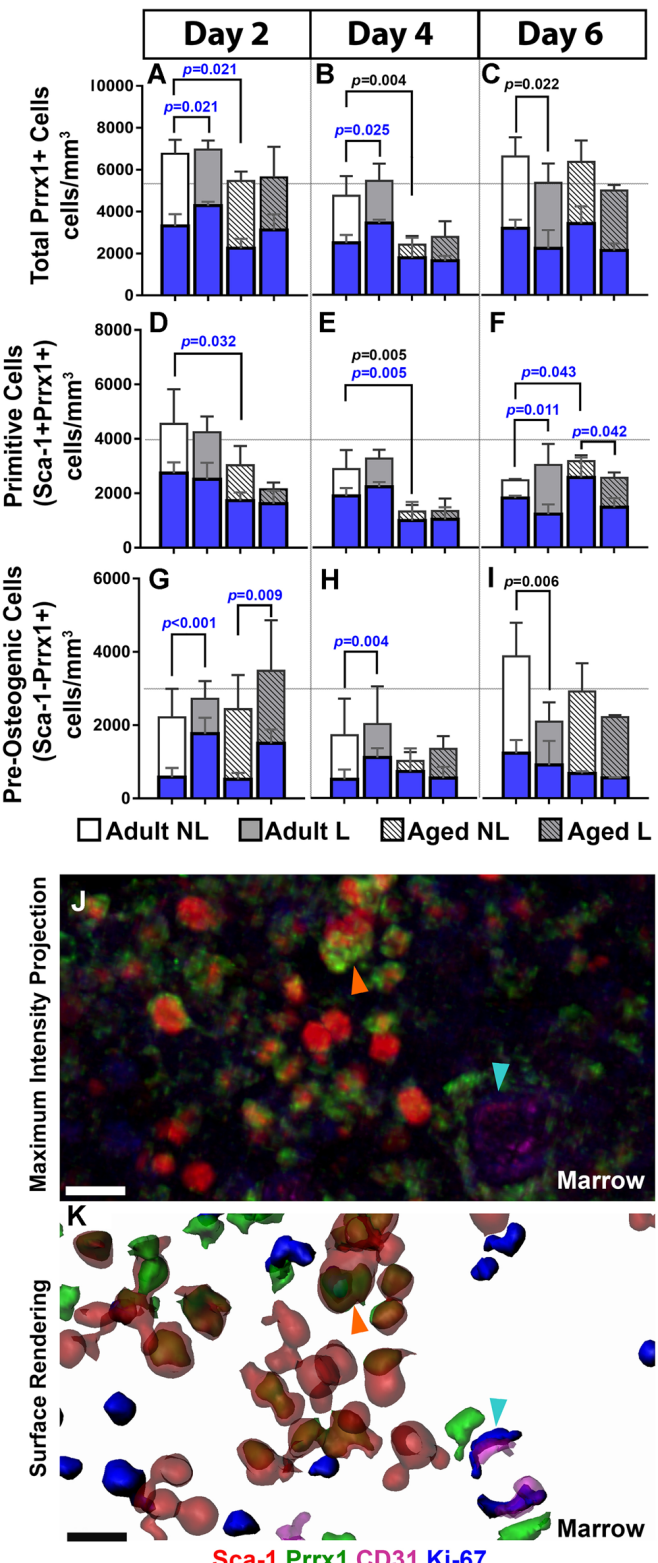
Quantification of marrow‐resident cell populations in adult and aged nonloaded (NL) and loaded (L) tibias using deep‐tissue immunohistochemistry. Total cell numbers are represented by white and gray bars, and proliferating cell number within each of these populations are represented by blue bars. (*A–C*) Total Prrx1 + , (*D–F*) Sca‐1^+^Prrx1^+^ and (*G–I*) Sca‐1^−^Prrx1^+^ cell number at days 2, 4, and 6. Representative image of the marrow from adult loaded bone displayed in a (*J*) maximum intensity projection and the corresponding (*K*) surface rendering showing Sca‐1 + (red), Prrx1 + (green), Ki‐67 + (blue), and CD31 + (magenta) cells. The marrow contains resting Sca‐1^+^Prrx1^+^ Ki‐67– cells (orange arrow) and proliferating CD31 + Ki‐67 + endothelial cells (aqua arrow). Significant differences between total cell number are indicated by *p* values shown in black. Significant differences between proliferating cell numbers are indicated by *p* values shown in blue. Scale bars = 5 µm.

Age‐associated changes in the marrow and the effects of mechanical loading on the resident stem cell population were investigated (Fig. *6A‐K*). At days 2 and 4, the number of proliferating Prrx1 + cells in loaded tibias increased significantly in adult (*p* = 0.021), but not aged mice relative to controls (Fig. 6A*,B*). At day 2, both adult (*p* < 0.001) and aged (*p* = 0.009) mice exhibited significant increases in proliferating Sca‐1^−^Prrx1^+^ cells in response to loading (Fig. 6 *G*), and this effect persisted in adult, but not aged mice on day 4 (Fig. 6 *H*). These data suggest that loading preferentially activates proliferation of Sca‐1^−^Prrx1^+^ cells in both adult and aged bone, with a more sustained effect in adult bone. This is in contrast to that observed at the periosteum and endosteum where the Sca‐1^+^Prrx1^+^ population is targeted by loading.

## Discussion

Mechanical loading initiates new bone formation and is a potent regulator of bone mass, though this beneficial response wanes as a function of age in both humans and preclinical models.^1^, ^4^, ^13^, ^36^, ^37^, ^45^ The central tenet of load‐induced bone formation is that mechanical deformation, or strain, activates osteoprogenitor proliferation^16^ and differentiation into bone‐forming osteoblasts,^16^, ^46^, ^47^, ^48^, ^49^ and that an age‐related decline in osteoprogenitor number and/or their ability to differentiate may account for an attenuated response to loading.^23^, ^50^, ^51^, ^52^ Our data reveal that both adult and aged mice can form new bone on the periosteal surface, accompanied by significant increases in cortical area and moments of inertia, in response to mechanical loading; this response is significantly attenuated with age, in agreement with data published by other groups.^1^, ^11^, ^12^, ^13^, ^15^, ^37^ Loading did not enhance bone formation on the endocortical surface in either age group. These results differ from those presented in previous studies, which show an increase in endosteal bone formation in adult C57BL/6 mouse tibias in response to loading.^13^, ^53^ This discrepancy may be explained by the relatively lower predicted peak strains at the endocortical surface^54^ compared with those measured at the periosteum because of its closer proximity to the neutral axis of bending and/or the differences in loading parameters (eg, frequency, waveform, rest insertion, etc.) across studies. For example, we use a 2‐Hz‐frequency sinusoidal waveform compared with a 4‐Hz‐frequency triangular waveform used by others.^13^, ^54^

*Prrx1*, a homeobox gene encoding a transcriptional regulator expressed in mesenchyme during embryogenesis^34^, ^55^ and in adult skeletal stem cells,^20^, ^21^ is required for skeletogenesis.^34^ Prrx1 has been identified as a suppressor of osterix and Runx2^56^ and an inhibitor of adipogenesis,^57^ thereby acting as a mediator of cellular stemness. Prrx1 + cells reside in the inner layer of the periosteum^20^, ^58^ and have been identified as osteochondral progenitors that contribute to osteogenesis during adult bone repair.^20^, ^21^, ^59^, ^60^ Thus, it follows that Prrx1 + cells may also play a key role in load‐induced osteogenesis considering their presence in the periosteum, the primary site of load‐induced cortical bone formation. Indeed, our deep‐tissue imaging shows that loading activates proliferation of Prrx1 + cells in the periosteum as early as 2 days into a 4‐consecutive‐day loading protocol, suggesting that expansion of the Prrx1 + population may be an important early event required for periosteal bone formation. Moreover, loading targets both the Sca‐1^+^Prrx1^+^ and Sca‐1^−^Prrx1^+^ cells^35^ at days 2 and 4, respectively, suggesting that different subpopulations of osteoprogenitor cells are activated at different times during the acute response to loading. These data support the findings of Turner and colleagues^16^ who showed that 60% to 90% of cells at the periosteal surface originated from proliferating cells, whereas > 95% of cells at the endocortical surface were recruited.

That expansion of the Prrx1 + population is absent in aged mice, even when similar numbers are present in adult and aged periosteum as assessed by deep‐tissue imaging, suggests there is an age‐related decline in their proliferative capacity, a common hallmark of cellular senescence.^52^ In addition, the more elongated nuclear shape observed in aged periosteum may be indicative of condensed chromatin and reflect functional changes in aged cells.^61^, ^62^ Notedly, there were fewer total cells in aged periosteum by DAPI staining, implying fundamental alterations in the periosteal stem cell niche, which might include changes in resident cell types, their relative numbers, and their potential to influence neighboring Prrx1 + cells via paracrine signaling.

Loading did not affect numbers or proliferative status of Prrx1 + cells at the endocortical surface relative to nonloaded controls at any experimental time point, but instead resulted in a temporal increase with significantly greater Prrx1 + cell numbers by day 6 versus days 2 and 4 in adult, but not aged loaded bone. These data suggest at least three possibilities: (1) that the proliferative response at the endocortical surface lags behind the periosteal response; (2) that bone‐forming osteoblasts at the endosteal surface are recruited from cells in the marrow or originate through differentiation of progenitors at the endocortical surface, which requires additional time; and/or (3) that newly expanded cells from the endosteal surface caused by loading are shuttled towards the periosteum through the vasculature. In a previous study comparing the proliferative response to mechanical loading at the periosteum and endosteum, greater than 95% of endocortical osteoblasts originated from nonproliferating cells 2 days after loading, whereas only 30% to 40% originated from nondividing osteoblasts at day 4. In contrast, almost 90% of periosteal osteoblasts originated from dividing cells at day 4.^16^ These findings together with our data suggest that loading preferentially enhances proliferation of periosteal‐resident osteoprogenitors, and recruitment to and/or differentiation of osteoprogenitors at the endocortical surface during the acute response phase, and that these load‐induced changes are attenuated with age.

Bone marrow contains multipotent mesenchymal stromal cells that have been identified by various overlapping markers including LepR,^25^ CXCL12,^26^ Sca‐1,^24^, ^27^ CD166,^24^ PDGFRa,^27^ Nestin,^28^ NG2,^29^ and Gremlin 1.^30^ Although there is evidence that mechanical loading enhances proliferation of precursors in the marrow,^16^ the contribution of distinct subpopulations to load‐induced bone formation is not well‐understood. Our data show that mechanical loading triggers proliferation of the Sca‐1^−^Prrx1^+^ population, but not the Sca‐1^+^Prrx1^+^ population, in adult bone marrow at days 2 and 4. Whether these cells ultimately differentiate into mature osteoblasts and contribute to new bone formation is under investigation. That aged bone exhibited a significant increase in proliferating Sca‐1^−^Prrx1^+^ cells in response to loading at day 2, but not by day 4, suggests that in an aged environment, osteoprogenitors have a shorter‐lived proliferative response to loading. Indeed, osteoprogenitor cells from aged bone exhibit decreased proliferative capacity in long‐term in vitro cell cultures relative to those from young healthy bone,^52^, ^63^ which is caused, in part, by the accumulation of DNA damage and increased cellular senescence with aging.^64^, ^65^ There are mixed results regarding the effects of aging on osteogenic differentiation of multipotent stromal cells,^23^, ^66^, ^67^, ^68^ and additional studies are required to determine whether load‐induced differentiation is also altered in an aging environment.

We expected to see a significant age‐related decline in Prrx1 + cells at all sites based on previous data showing decreases in the number of mesenchymal stem cells in aged bone.^31^, ^63^, ^69^ Accordingly, we observed significantly lower numbers of total and proliferating Sca‐1^+^Prrx1^+^ in the periosteum and marrow in nonloaded aged versus adult bone, primarily at days 2 and 4, suggesting that the niche's ability to maintain the Sca‐1^+^Prrx1^+^ population wanes with age. Indeed, others have shown that osteoprogenitors are localized in close proximity to specialized H‐type vessels, so named for their high levels of endomucin and CD31 expression. H‐type vessels are located primarily near the endocortical surface and below the growth plate in growing mouse bone, but experience a significant reduction in number with aging.^23^, ^70^ This decline in H‐type vessels corresponds with a decline in Osx + cells, a more differentiated osteogenic cell, and an increase in marrow fat.^71^, ^72^ Interestingly, we did not see a decline in Sca‐1^+^Prrx1^+^ cells at the endocortical surface with age, suggesting that Sca‐1^+^Prrx1^+^ cells may localize to structures other than H‐type vessels at the endocortical surface.

There are important strengths and limitations of this study. Previous studies have used double fluorochrome bone labels and immunohistological staining of thin sections to track bone‐forming surfaces, osteoblast activity, and cellular proliferation in response to mechanical loading.^8^, ^16^, ^73^, ^74^ These approaches cannot rigorously assess activation of individual stem cell populations in distinct 3D skeletal niches. Our use of deep‐tissue imaging allows 3D visualization, identification, and quantification of subpopulations of osteoprogenitors in three skeletal compartments over time during the acute response phase. This approach allows comparisons at the cellular level between adult and aged bone. One limitation of this study is that analyses of the periosteal and endosteal surfaces were limited to a small region because of limited antibody penetration into the sample. Because of this, we chose to look at the medioanterior aspect of the tibia where there is little musculature coverage. A second limitation is the absence of a day 0 time point, which limits our ability to evaluate the loading response at day 2 against a control. However, we do provide appropriate controls for assessing effects of aging and loading; therefore, we do not consider this a major omission. A third limitation of fixed tissue analyses is the static snapshot of what is a dynamic biological process; for this reason, we examined bone tissue at three time‐points to detect cell‐population changes. Furthermore, whether osteoprogenitor cells in long bone are of the same type as those present in sutures of the cranium remains unanswered in this present work. Even though both load‐induced bone formation of long bones and bone apposition of the cranium occurs by intramembranous ossification, our studies were limited to long bones.

In summary, loading tends to activate the Sca‐1^+^Prrx1^+^ cell population to proliferate at the periosteal and endocortical surfaces, and the Sca‐1^−^Prrx1^+^ population in the marrow in adult bone. Aged bone displays an attenuated, though not absent, osteogenic response at the periosteal and endosteal surfaces, which may be caused by fewer Prrx1 + cells and a diminished proliferative response of the Sca‐1^+^Prrx1^+^ population. In contrast, loading enhances proliferation of Sca‐1^−^Prrx1^+^ cells in the marrow in both adult and aged bone, an effect that is maintained for a longer period in younger bones. Accordingly, though mechanical loading presents an attractive inexpensive treatment for increasing bone mass, exercise alone may not be enough to prevent or reverse age‐related bone loss caused by the decline in stem cell responsiveness. Therefore, a successful strategy for prevention and treatment of osteoporosis will be one that preserves stem cell number and uses both osteoanabolics and mechanical loading to drive cellular proliferation and osteogenic differentiation in areas of low bone mass.

## Disclosures

The authors declare no competing financial interests.

## Supporting information

Supporting Information.
