# Supplementary material for: Site‐Specific Load‐Induced Expansion of Sca‐1+Prrx1+ and Sca‐1−Prrx1+ Cells in Adult Mouse Long Bone Is Attenuated With Age
Source: JBMR Plus. 2019 Jul 30;3(9):e10199. doi: 10.1002/jbm4.10199 (PMC6808224; doi:10.1002/jbm4.10199)
Supplement: Supplementary file 1 — Supporting Information. [file JBM4-3-na-s001.docx]

**Supplemental Information**

**Deep Tissue Immunohistochemistry**

**Table S1.** *Deep Tissue Immunohistochemistry Primary Antibodies*

| **Antigen** | **Company** | **Catalog number** | **Reactivity** | **Species** |
| --- | --- | --- | --- | --- |
| Ki67 | R&D | AF7649-SP | Mouse | Sheep |
| Prrx1 | Abcam | ab211292 | Mouse | Rabbit |
| Sca1 | e-Bioscience | 14-5981-82 | Mouse | Rat |
| CD31 | Santa Cruz | sc-1506 | Mouse | Goat |

**Table S2.** *Deep Tissue Immunohistochemistry Secondary Antibodies*

| **Conjugate** | **Company** | **Catalog number** | **Reactivity** | **Species** |
| --- | --- | --- | --- | --- |
| Alexa Fluor 405 | Abcam | ab175676 | Sheep | Donkey |
| Alexa Fluor 488 | Fisher Scientific | A-21206 | Rabbit | Donkey |
| Alexa Fluor 594 | Fisher Scientific | A-21209 | Rat | Donkey |
| Alexa Fluor 647 | Fisher Scientific | A-21447 | Goat | Donkey |

**Table S3.** *Blocking Solutions*

| **Name** | **Company** | **Catalog number** |
| --- | --- | --- |
| Normal donkey serum | Abcam | Ab7475 |
| Bovine serum albumin | Fisher Scientific | BP675-1 |
| Normal sheep IgG | R&D | 5-001-A |
| Normal rabbit IgG | R&D | AB-105-C |
| Normal rat IgG | R&D | 6-001-A |
| Normal goat serum | Cell Signaling | 5425 |

**Imaris Surface Rendering Parameters**

**Table S4.** *High-Resolution Image Acquisition*

| **Target** | **Fluorophore** | **Laser (nm)** | **% Intensity** | **Emission Bandwidth** | **Track** |
| --- | --- | --- | --- | --- | --- |
| Ki67 | Alexa Fluor 405 | 405 | 5 | 420-460 | 1 |
| Prrx1 | Alexa Fluor 488 | 488 | 7 | 510-540 | 2 |
| Sca1 | Alexa Fluor 594 | 544 | 30 | 610-650 | 1 |
| CD31 | Alexa Fluor 633 | 633 | 15 | 660-700 | 2 |

*Note*. For all channels, Gain = 650, Pinhole size = 32.6 µm (Airy Unit =1.03 – 1.23), 20X Water Objective (NA 1.0), x-y pixel resolution = 0.4 µm, z-interval = 1 µm

**Table S5.** *Parameters for Image Analyses Using Imaris Software*

| **Target** | **Low Threshold** | **Largest sphere fit (µm)** | **Estimated diameter (µm)** | **Sphericity** | **Volume Range (µm^3^)** |
| --- | --- | --- | --- | --- | --- |
| Ki67 | 4-7 | 5 | 4 | 0.2 - 0.99 | 64-500 |
| Prrx1 | 6-8 | 5 | 4 | 0.2 - 0.99 | 64-500 |
| Sca1 | 8-15 | 6 | 5 | 0.3 - 0.99 | 125-800 |
| CD31 | 2-5 | 6 | 5 | 0.3 - 0.99 | 125-800 |

*Note.* For all channels, smoothing size = 1 µm, surface rendering quality = 1-10

**Table S6.** *Calibration of Cell Quantification: Comparison of Manual and Automatic Methods*

|  | Automatic | | | | Manual | | | | Mean Δ (%) |
| --- | --- | --- | --- | --- | --- | --- | --- | --- | --- |
| **ID** | **Prrx1+** | **Prrx1+ Sca-1+** | **Prrx1+ Sca-1+ Ki67+** | **Prrx1+ Ki67+** | **Prrx1+** | **Prrx1+ Sca-1+** | **Prrx1+ Sca-1+ Ki67+** | **Prrx1+ Ki67+** |  |
| 74L | 133 | 22 | 12 | 41 | 121 | 21 | 11 | 44 | 7 |
| 75L | 119 | 27 | 25 | 37 | 129 | 25 | 24 | 36 | 6 |
| 76L | 85 | 20 | 26 | 36 | 91 | 20 | 28 | 32 | 6 |
| 77L | 117 | 32 | 18 | 56 | 116 | 29 | 19 | 54 | 5 |
| 78L | 56 | 25 | 17 | 49 | 56 | 22 | 16 | 48 | 5 |
| 79L | 98 | 21 | 15 | 59 | 102 | 19 | 14 | 59 | 5 |
| 74R | 121 | 27 | 27 | 81 | 122 | 28 | 25 | 76 | 5 |
| 75R | 61 | 25 | 24 | 47 | 62 | 24 | 22 | 44 | 5 |
| 76R | 172 | 41 | 33 | 85 | 175 | 36 | 32 | 90 | 6 |
| 77R | 97 | 40 | 31 | 80 | 98 | 37 | 34 | 81 | 5 |
| 78R | 155 | 28 | 26 | 82 | 179 | 30 | 25 | 83 | 7 |
| 79R | 81 | 20 | 17 | 60 | 85 | 20 | 17 | 60 | 1 |

*Note.* % Mean absolute difference (Δ) between manual and automatic cell counts derived from the periosteal envelope of adult day 4 left (L) non-loaded and right (R) loaded tibiae.

**Table S7.** *Comparison of periosteal (Ps) and endosteal (En) osteoprogenitor numbers in non-loaded control limbs at day 2 of described loading protocol (see Methods) in adult and aged mice.*

|  | Adult (n=6) | | | Aged (n=4) | | |
| --- | --- | --- | --- | --- | --- | --- |
| **Cell Population** | **Ps** | **En** | ***p-value*** | **Ps** | **En** | ***p-value*** |
| Total Prrx1+ (#/mm^2^) | 518 ± 97 | 653 ± 169 | 0.5047 | 467 ± 121 | 467 ± 80 | 0.9987 |
| Proliferating**^a^** Prrx1+ (#/mm^2^) | 90 ± 16 | 184 ± 38 | 0.0464* | 95 ± 24 | 114 ± 26 | 0.6078 |
| Total Sca-1+Prrx1+ (#/mm^2^) | 51 ± 8 | 148 ± 40 | 0.0412* | 46 ± 11 | 122 ± 39 | 0.1088 |
| Proliferating**^a^** Sca-1+Prrx1+ (#/mm^2^) | 15 ± 5 | 76 ± 20 | 0.0164* | 15 ± 4 | 44 ± 11 | 0.0485 |
| Total Sca-1-Prrx1+ (#/mm^2^) | 468 ± 92 | 505 ± 132 | 0.8199 | 420 ± 116 | 344 ± 60 | 0.5818 |
| Proliferating**^a^** Sca-1-Prrx1+ (#/mm^2^) | 75 ± 19 | 108 ± 20 | 0.2602 | 80 ± 20 | 70 ± 17 | 0.7121 |

*Note.* Cell numbers are normalized to bone surface area (mm^2^) from which the periosteal and endosteal volumes were derived.

Data expressed as means ± SD.

* p < 0.05 by Student’s t-test.

**^a^**Ki-67+ co-expression.

**
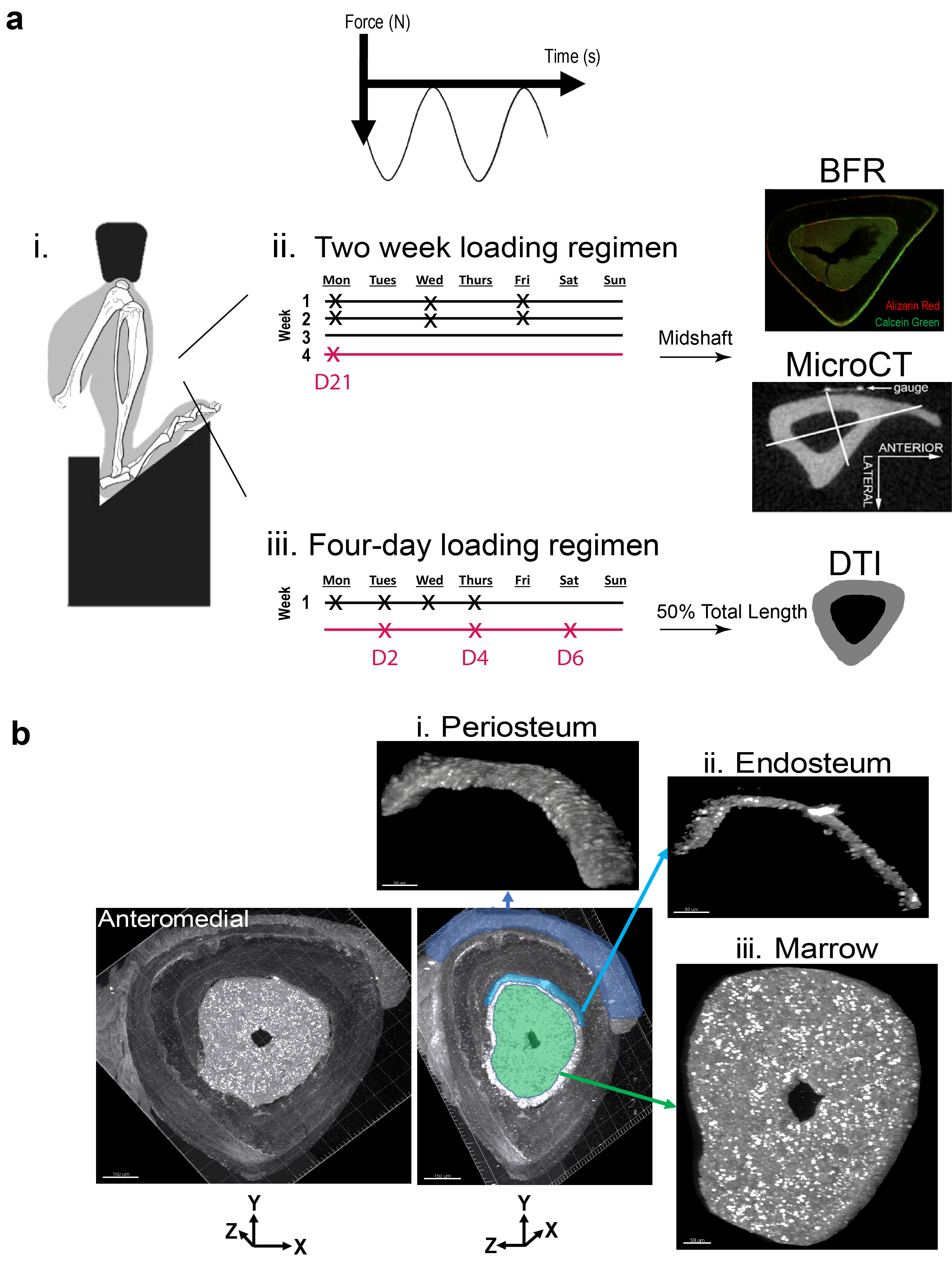
**

**Figure S1. (a)** Experimental design, including an illustration of the (i) tibial axial compression model and waveform, the timeline for a (ii) two-week, where tibia were harvested at Day 21 (D21) and (iii) four-day loading regimen, where tibiae were harvested at Days 2 (D2), 4 (D4) or 6 (D6), and their corresponding output readouts; i.e. dynamic histomorphometry to assess bone formation rates (BFR), microCT to assess bone microarchitecture, and deep tissue immunohistochemistry (DTI) to assess cell populations, respectively. (b) Schematic of the volumes of interest used to analyze cell populations from transversely cut thick bone sections. These volumes of interests and range of volumes examined are (i) periosteum: 5-15 x 10^6^ µm^3^, (ii) endosteum: 2-10 x 10^6^ µm^3^ and (iii) marrow: 10-30 x 10^6^ µm^3^.

**
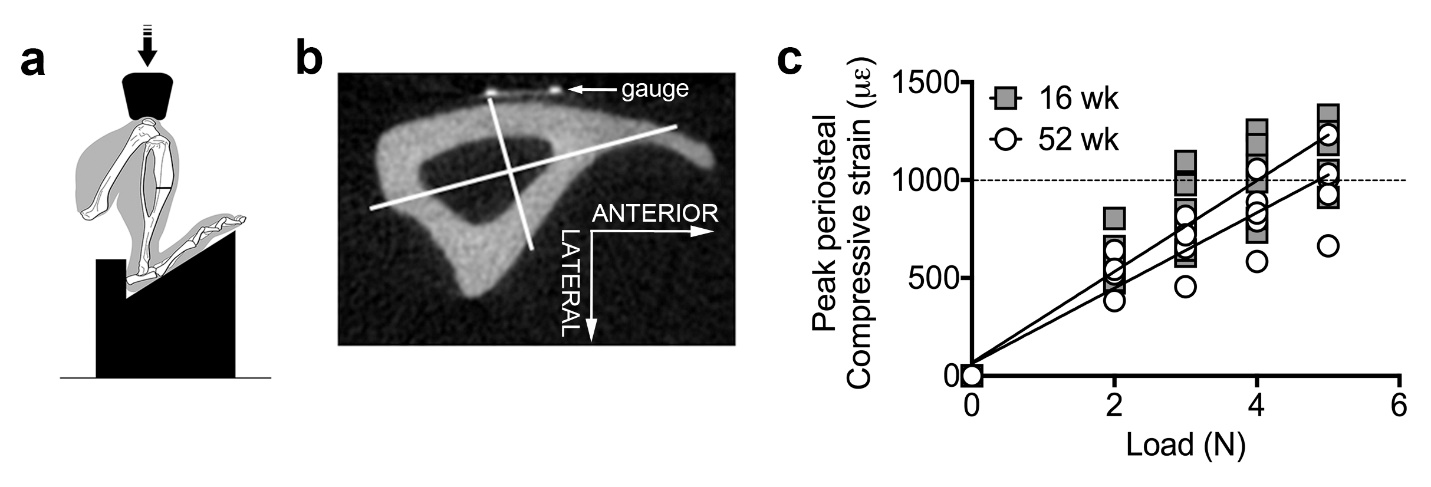
**

**Figure S2.** *In vivo* tibial axial compression model and load-strain calibration curves. (a) Schematic of the mouse tibia secured at the knee and ankle in the loading configuration. (b) Cross-sectional CT image of the tibia showing the strain gauge location and the major and minor axes about the centroid about which the maximum and minimum area moment of inertia are calculated. (c) Load-strain calibration data presented as individual data points with a line of best fit for adult and aged mice.

**
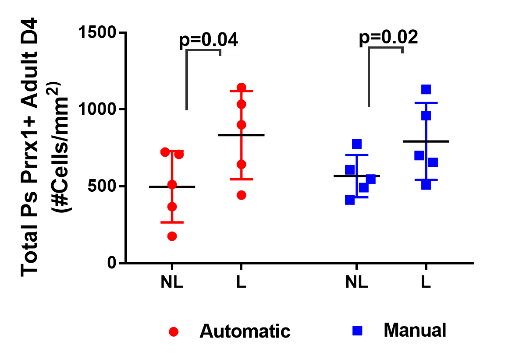
**

**Figure S3.** Comparison between automatic (red) and manual (blue) quantification. Total periosteal Prrx1+ cells from adult (n=5) non-loaded (NL) and loaded (L) tibiae of the day 4 (D4) group described in Methods, were quantified using the Imaris surface rendering cell counting functionality, automatically and manually. Paired Student’s t-test, with alpha = 0.05 was used to compare NL and L values within the same method used (either Automatic or Manual), and 2-way ANOVA to compare Automatic and Manual values. Significance of p < 0.05 is indicated in the graph.

**
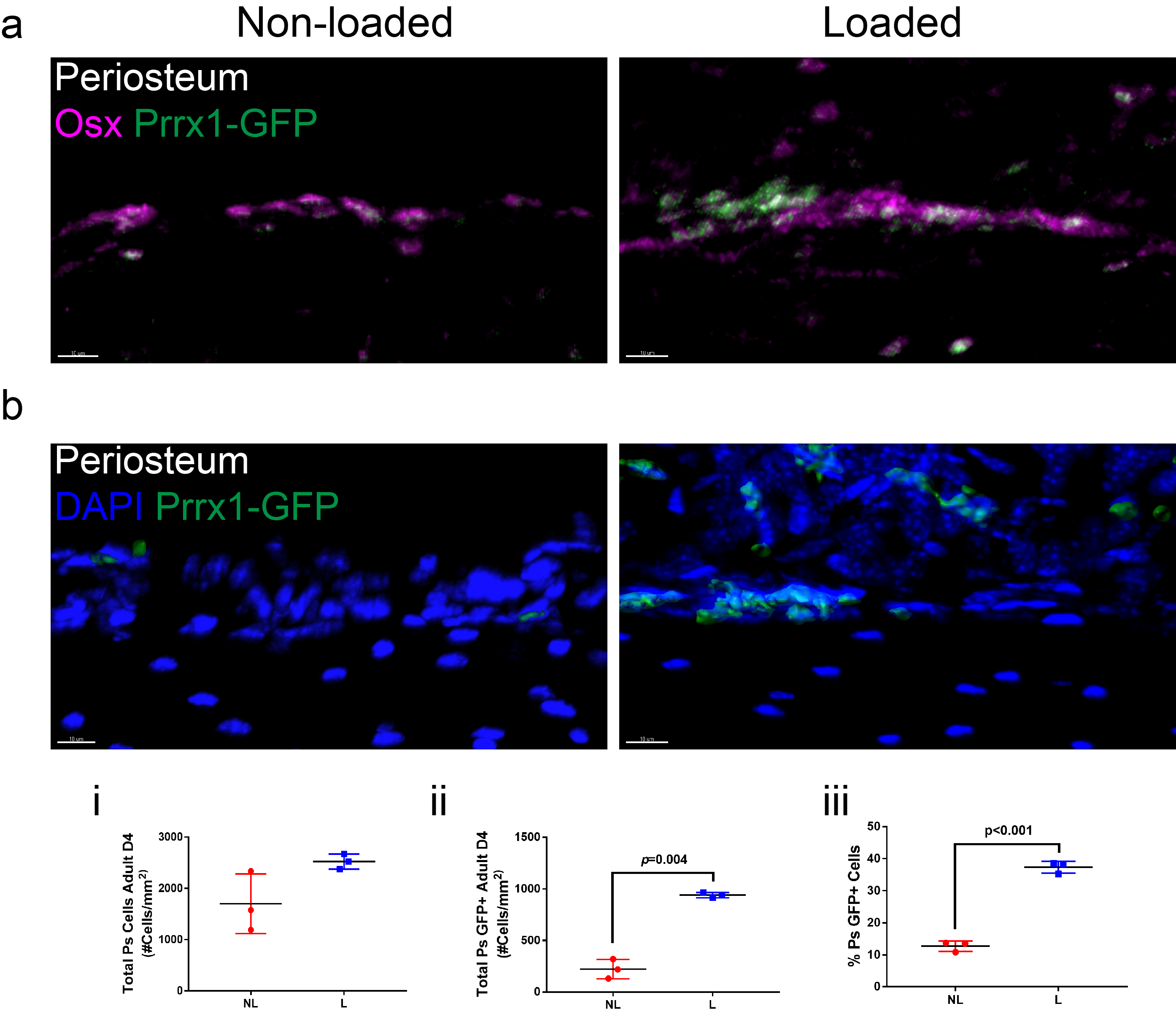
**

**Figure S4. (a)** Representative images showing localization of Osx+ (magenta) cells and cells expressing Prrx1-eGFP (green) from the periosteum of non-loaded and loaded 19-week-old animals. **(b)** Representative images used to quantify (i) total number of cells, (ii) Prrx1-eGFP expressing cells and (iii) the percentage of GFP+ cells, from the periosteum. Right tibiae of mice (n=3) were subjected to mechanical loading (L) with 6 N Haversine, 120 cycles, 2 Hz daily for four days, while the left limbs were non-loaded (NL) contralateral controls. Tibiae were harvested and processed for deep tissue immunohistochemistry, as described in Methods. Transverse thick cryo-sections from the 50% tibial length region were DAPI stained, mounted, and imaged with a Zeiss LSM710 to detect GFP+ and DAPI staining. Periosteal volumes of interest were analyzed automatically for DAPI+ and GFP+ cell numbers. Total cell numbers (DAPI), GFP^+^, and % of GFP^+^/Total Cells, are shown, respectively. Paired Student’s t-test, with alpha = 0.05 was used to compare NL and L values. Significance of p < 0.05 is indicated in the graph.

**
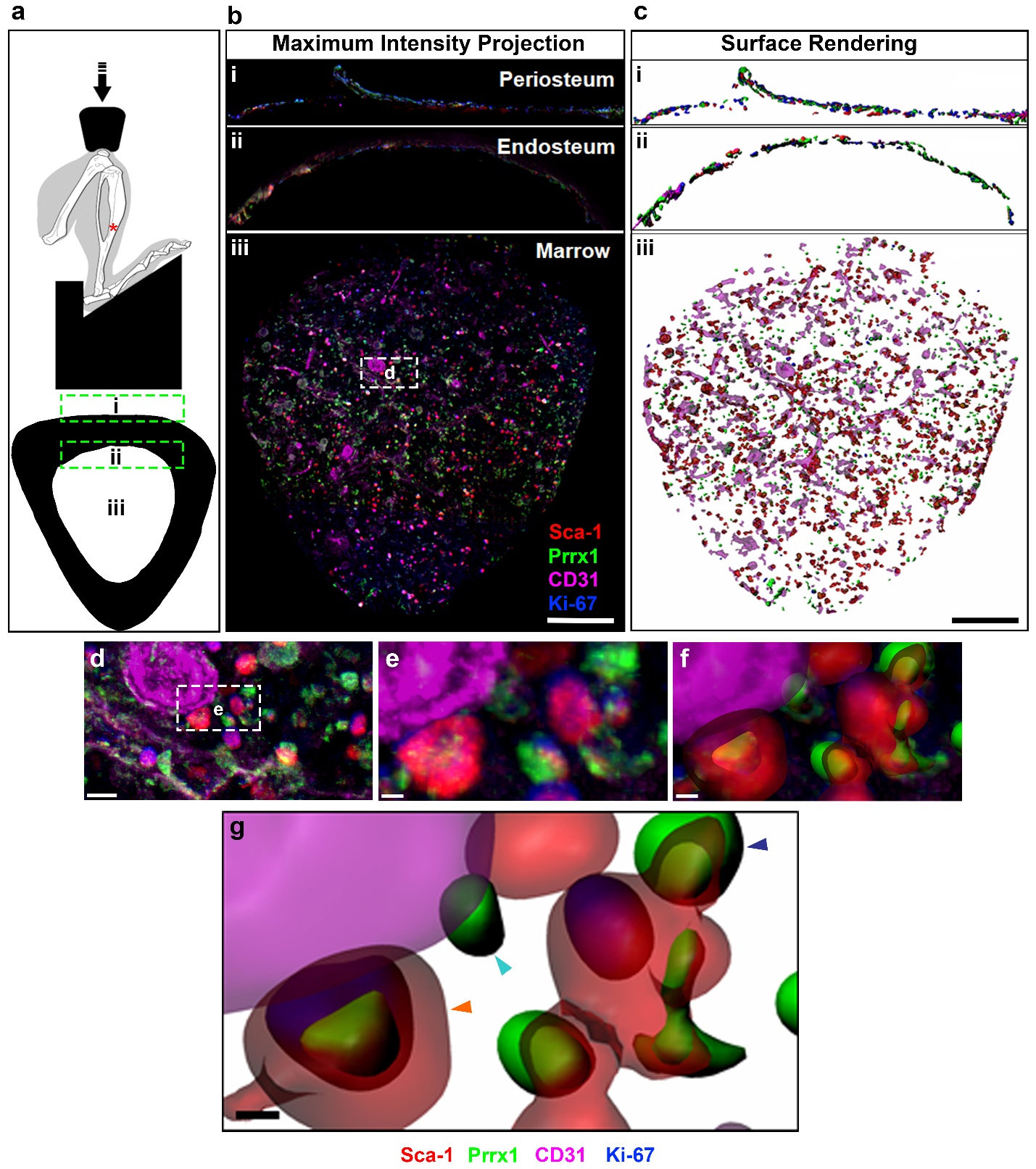
**

**Figure S5.** Single-cell detection of Sca-1+Prrx1- and Sca-1-Prrx1-cells using deep tissue immunohistochemistry. (a) Thick (150 µm) transverse sections at the tibial mid-shaft (red*) were immunostained for Sca-1 (red), Prrx1 (green), Ki-67 (blue) and CD31 (magenta). Resulting confocal image volumes were divided into (i) periosteal, (ii) endosteal and (iii) marrow regions and visualized as (b) maximum intensity projections (MIP) and (c) surface renderings (SR). (b) Representative MIP images of the (i) periosteum, (ii) endosteum and (iii) marrow, and the corresponding (c) SR images by Imaris v7.4.2. Manual thresholding was used to identify positively stained cells based on size and morphology. For membrane-bound proteins Sca-1 and CD31, the allowed range of values for volume (V) was 200 to 1200 µm^3^, and the diameter (D) was 6 to 10 µm; for nuclear proteins Prrx1 and Ki-67, V was 125 to 1000 µm^3^ and D was 5-6 µm. For all channels, the range of sphericity was 0.3 to 0.95. (d) Magnified image of an area (white dotted line) within the marrow shown in b-iii. (e) Magnified image of an area (white dotted line within (d). (f) MIP and SR combined. (g) Proliferating Sca-1+Prrx1+Ki-67+ cell (orange arrow), a resting Sca-1+Prrx1-Ki-67- cell (purple arrow) and resting Sca-1-Prrx1+Ki-67- cell (aqua arrow). Scale bars = 100 µm in b,c; 5 µm in d; and 2 µm in e-g.
